# C-myc amplification in breast cancer: a meta-analysis of its occurrence and prognostic relevance

**DOI:** 10.1054/bjoc.2000.1522

**Published:** 2000-12

**Authors:** S L Deming, S J Nass, R B Dickson, B J Trock

**Affiliations:** Department of Oncology and Lombardi Cancer Center, Georgetown University, Wisconsin Avenue, Washington, DC, 20007; Oncology Center, The Johns Hopkins University School of Medicine, Baltimore, MD, 21231, USA

**Keywords:** *c-myc*, amplification, breast cancer, prognostic markers, meta-analysis

## Abstract

Data from basic research suggests that amplification of the proto-oncogene *c-myc* is important in breast cancer pathogenesis, but its frequency of amplification and prognostic relevance in human studies have been inconsistent. In an effort to clarify the clinical significance of *c-myc* amplification in breast cancer, we conducted a comprehensive literature search and a meta-analysis in which 29 studies were evaluated. The weighted average frequency of *c-myc* amplification in breast tumours was 15.7% (95% CI = 12.5–18.8%), although estimates in individual studies exhibited significant heterogeneity, *P*< 0.0001. *C-myc* amplification exhibited significant but weak associations with tumour grade (RR = 1.61), lymph-node metastasis (RR = 1.24), negative progesterone receptor status (RR = 1.27), and postmenopausal status (RR = 0.82). Amplification was significantly associated with risk of relapse and death, with pooled estimates RR = 2.05 (95% CI = 1.51–2.78) and RR = 1.74 (95% CI = 1.27–2.39), respectively. This effect did not appear to be merely a surrogate for other prognostic factors. These results suggest that *c-myc* amplification is relatively common in breast cancer and may provide independent prognostic information. More rigorous studies with consistent methodology are required to validate this association, and to investigate its potential as a molecular predictor of specific therapy response. © 2000 Cancer Research Campaign http://www.bjcancer.com


*British Journal of Cancer* (2000) **83**(12), 1688–1695
© 2000 Cancer Research Campaign
doi: 10.1054/ bjoc.2000.1522, available online at http://www.idealibrary.com on IDEAL®

http://www.bjcancer.com

### C-myc amplification in breast cancer: a meta-analysis of its occurrence and prognostic relevance

**SL Deming[1], SJ Nass[2], RB Dickson[1] and BJ Trock[1]**

[1]Department of Oncology and Lombardi Cancer Center, Georgetown University, Wisconsin Avenue, Washington, DC 20007; [2]Oncology Center, The Johns Hopkins University School of Medicine, Baltimore, MD 21231, USA

**Summary** Data from basic research suggests that amplification of the proto-oncogene *c-myc* is important in breast cancer pathogenesis, but its frequency of amplification and prognostic relevance in human studies have been inconsistent. In an effort to clarify the clinical significance of *c-myc* amplification in breast cancer, we conducted a comprehensive literature search and a meta-analysis in which 29 studies were evaluated. The weighted average frequency of *c-myc* amplification in breast tumours was 15.7% (95% CI = 12.5–18.8%), although estimates in individual studies exhibited significant heterogeneity, $P < 0.0001$. *C-myc* amplification exhibited significant but weak associations with tumour grade (RR = 1.61), lymph-node metastasis (RR = 1.24), negative progesterone receptor status (RR = 1.27), and postmenopausal status (RR = 0.82). Amplification was significantly associated with risk of relapse and death, with pooled estimates RR = 2.05 (95% CI = 1.51–2.78) and RR = 1.74 (95% CI = 1.27–2.39), respectively. This effect did not appear to be merely a surrogate for other prognostic factors. These results suggest that *c-myc* amplification is relatively common in breast cancer and may provide independent prognostic information. More rigorous studies with consistent methodology are required to validate this association, and to investigate its potential as a molecular predictor of specific therapy response. © 2000 Cancer Research Campaign http://www.bjcancer.com

**Keywords:** *c-myc*; amplification; breast cancer; prognostic markers; meta-analysis

There is mounting evidence to support a role for the *c-myc* proto-oncogene in tumour onset and progression. Myc directly modulates the basal transcription apparatus, as well as specific genes containing the consensus E-box element. Myc-responsive genes include those whose protein products regulate the cell cycle and cell death. Abnormal regulation of the *c-myc* gene by multiple mechanisms can result in phenotypic transformation, aberrant cell cycle control, and genomic instability. However, at present, only its gene amplification and overexpression have been described in breast cancer; neither gene rearrangement nor mutation have been described. No clear relationships have yet been described between *c-myc* amplification and overexpression of its mRNA nor protein in breast cancer nor in other tumour types where *c-myc* is commonly amplified (Varmus, 1984; Nass and Dickson, 1997; Dang, 1999).

In breast cancer, the chromosome 8 region where the gene is localized has been identified as one of the three most commonly amplified regions of the genome (Courjal et al, 1997); this region also is commonly amplified in small cell lung carcinoma (Little et al, 1985), leukaemia (Dalla-Farera et al, 1985), and colon carcinoma (Alitalo et al, 1983). *C-myc* has been considered to be the dominant oncogene in this region, driving the selection of this amplification in cancer. This supposition is based on the common expression of *c-myc* coupled with many demonstrations of its oncogenic properties in multiple types of cancer models. For example, in mouse transgenic models expression of the transgene under either the MMTV or WAP promoter, or by retroviral strategies, resulted in mammary tumours following multiple pregnancies (Leder et al, 1986; Edwards et al, 1988).

These observations suggest that anomalous expression of *c-myc* may bring about a cascade of effects: altered cell cycle progression, genomic instability, and in some instances, tumorigenesis. In breast cancer, investigation of the relationship between the biological function and the clinical implications of *c-myc* gene amplification has produced inconsistent results. In an attempt to clarify the clinical relevance of *c-myc* amplification in breast cancer we performed a meta-analysis of the literature. To achieve this goal, our meta-analysis set out to address three specific questions:

1. What is the usual frequency of *c-myc* amplification in breast tumours?
2. Which prognostic factors are significantly associated with *c-myc* amplification, and how strong are the associations?
3. To what degree is *c-myc* amplification in breast tumours associated with disease relapse and/or survival?

### MATERIALS AND METHODS

### Source of articles and methods of citation search

Articles evaluating *c-myc* amplification in human breast tumours were identified through a literature search of the following databases: Medline (National Library of Medicine, National Institutes of Health, Bethesda, MD, USA), Current Contents (Institute for Scientific Information, Philadelphia, PA, USA) and PubMed. *Knowledge Finder* (Aries Systems Corp, North Andover, MA, USA) was utilized as a search engine, with 'amplification', 'oncogene', 'alteration', 'mutation', 'cancer', and 'breast' used as keywords for the search. Review articles and bibliographies from

*Received 10 February 2000*
*Revised 22 August 2000*
*Accepted 24 August 2000*

*Correspondence to:* BJ Trock relevant papers were also used as means of identifying additional studies.

### Inclusion and exclusion criteria

For inclusion in this meta-analysis, studies had to describe research determining the frequency of amplification of the *c-myc* gene in human breast tumours, and provide the number of amplified and non-amplified tumours. Studies that did not differentiate between gene amplification and other types of alteration (rearrangement and deletions) were excluded from the analysis. Multiple reports from the same study contributed only one estimate to the calculation of overall *c-myc* amplification frequency (Berns et al, 1992; Champeme et al, 1994; Scorilas et al, 1995). However, subsequent reports containing new data on prognostic factors or survival were also incorporated into pooled analyses of the specific clinical end-points. Data from studies that used more than one method to determine the overall frequency of amplification were included separately in subgroup analyses of the individual methods (Zhou et al, 1989; Watson et al, 1993). Consideration of methodologic aspects included the sources of tissue and amplification controls, specification of the positive threshold for gene amplification, and a description of the characteristics of the study population (Trock et al, 1997).

For the association between *c-myc* amplification and a prognostic factor to be analysed, at least three studies were required to report data concerning that factor. Likewise, at least three studies containing hazard estimates or raw data to generate survival estimates were required for the association between survival and *c-myc* amplification to be examined. This number is arbitrary and was thought to represent the minimum amount of data that could provide an informative estimate of the magnitude of an association.

### Methods of statistical analysis

For each study the proportion of breast tumours with amplified *c-myc* was extracted and 95% confidence intervals were calculated by standard methods (Fleiss, 1981). For a pooled measure of *c-myc* gene amplification frequency, the proportions from each study were combined in a weighted average, using study sample size as weights. Homogeneity of the proportions across studies was determined using a chi-squared test for contingency tables. (Fleiss, 1981).

The association of *c-myc* amplification with individual prognostic factors was expressed as a rate ratio (RR), i.e. the relative probability of *c-myc* amplification in tumours with the adverse prognostic factor compared to those without the adverse factor. Study-specific RRs were pooled across all studies with a Mantel-Haenszel approach (MH), using the procedure PROC FREQ in SAS (Statistical Analysis Systems Institute Inc, Cary, NC). The homogeneity of the RRs across the studies was determined using the method of Breslow and Day (1987).

The association between *c-myc* amplification and either overall survival (OS) or disease-free survival (DFS) was derived as a weighted average of study-specific estimates of the hazard ratio (HR), using inverse variance weights (Kleinbaum et al, 1982). This required the HR and its standard error (Tsuda et al, 1989; Berns et al, 1992; Borg et al, 1992), or inclusion of sufficient raw data in the published report for us to perform a multivariable proportional hazards regression (Varley et al, 1987) (using PROC PHREG in SAS). For studies that did not provide HR estimates or the raw data (Yamashita et al, 1993; Lonn et al, 1995), we derived estimates of the HRs by calculating the smallest HR that could be detected with power = 0.80 at the *P*-values actually observed, using standard power calculations for survival analysis (George and Desu, 1974). The standard error of this derived HR estimate was obtained by dividing the log (HR) estimate by the square-root of the Wald chi-square statistic associated with the observed *P*-value. As an ad hoc check on the validity of this approach we used the same derivation in the three studies that did provide HRs. The derived HRs from these three studies were in most instances conservative, that is, they were generally smaller than those actually observed in the studies.

### RESULTS

### Results of citation search

Forty reports were initially identified by our search. Eleven studies that reported only data concerning over-expression of the *c-myc* protein, or that did not distinguish between amplification and other alterations of the *c-myc* gene were excluded. The analysis included 29 reports (representing 26 distinct studies) that met the inclusion criteria. Twenty-six of the 29 reports were included in the estimation of the overall proportion of *c-myc*-amplified tumors (Escot et al, 1986; Cline et al, 1987; Varley et al, 1987; Bonilla et al, 1988; Guerin et al, 1988; Adnane et al, 1989; Garcia et al, 1989; Tavassoli et al, 1989; Tsuda et al, 1989; Zhou et al, 1989; Brouillet et al, 1990; Meyers et al, 1990; Munzel et al, 1991; Berns et al, 1992*b*; Borg et al, 1992; Roux-Dosseto et al, 1992; Kreipe et al, 1993; Nagayama and Watatani, 1993; Ottestad et al, 1993; Scorilas et al, 1993; Watson et al, 1996; Yamashita et al, 1993; Bieche et al, 1994; Harada et al, 1994; Contegiacomo et al, 1995; Lonn et al, 1995). Amplification data in Champeme et al (1994), Scorilas et al (1995) and Berns et al (1992) were excluded from estimation of the overall proportion, since they were originally reported in Bieche et al (1994), Scorilas et al (1993) and Berns et al (1992*b*), respectively. Of the 29 reports, 26 determined *c-myc* amplification by Southern blot, three by slot blot (Tsuda et al, 1989; Zhou et al, 1989; Borg et al, 1992), and two by polymerase chain reaction (Watson et al, 1993; Lonn et al, 1995). Two studies utilized more than one method (Zhou et al, 1989; Lonn et al, 1995).

Seven prognostic factors were analysed for their association with *c-myc* amplification: lymph node involvement (Escot et al, 1986; Cline et al, 1987; Varley et al, 1987; Adnane et al, 1989; Tavassoli et al, 1989; Berns et al, 1992*a*; Borg et al, 1992; Kreipe et al, 1993; Nagayama and Watatani, 1993; Scorilas et al, 1993; Champeme et al, 1994; Harada et al, 1994; Lonn et al, 1995), oestrogen receptor status (Escot et al, 1986; Varley et al, 1987; Adnane et al, 1989; Garcia et al, 1989; Berns et al, 1992*b*; Borg et al, 1992; Kreipe et al, 1993; Yamashita et al, 1993), progesterone receptor status (Escot et al, 1986; Adnane et al, 1989; Garcia et al, 1989; Berns et al, 1992*b*; Borg et al, 1992; Kreipe et al, 1993; Champeme et al, 1994), age (Escot et al, 1986; Guerin et al, 1988; Adnane et al, 1989; Berns et al, 1992*a*; Borg et al, 1992; Kreipe et al, 1993), menopausal status (Varley et al, 1987; Berns et al, 1992*a*; Borg et al, 1992; Yamashita et al, 1993), tumour size (Guerin et al, 1988; Adnane et al, 1989; Nagayama and Watatani, 1993; Yamashita et al, 1993), and tumour grade (Escot et al, 1986; Varley et al, 1987; Adnane et al, 1989; Garcia et al, 1989; Tavassoli et al, 1989; Kreipe et al, 1993). Data on the HR for death

© 2000 Cancer Research Campaign

**Table 1** Description of studies and methodologic aspects

| | Study size ($n$) | Frequency of *c-myc* amp (%) | 95% CI | Method | Normal DNA / amplification controls | Threshold for *c-myc* amplification | Description of study population |
|---|---|---|---|---|---|---|---|
| Escot et al, 1986 | 121 | 31.4 | 23.4–39.7 | Southern | PBLs / β-globin | 2 | – |
| Cline et al, 1987 | 53 | 15.1 | 7.2–26.0 | Southern | normal breast / β-globin | 3 | + |
| Varley et al, 1987 | 41 | 17.1 | 7.7–29.9 | Southern | PBLs, normal breast / HRPT gene | 2 | – |
| Bonilla et al, 1988 | 48 | 50.0 | 35.4–62.6 | Southern | PBLs / c-mos, β-tubulin | 2 | – |
| Guerin et al, 1988 | 100 | 6.0 | 2.5–11.8 | Southern | PBLs / β-globin 1 | 3 | + |
| Adnane et al, 1989 | 212 | 17.9 | 13.1–23.4 | Southern | not specified / c-mos, β-globin | 2 | + |
| Garcia et al, 1989 | 125 | 17.6 | 11.6–24.8 | Southern | PBLs / b-globin, c-mos | 2 | – |
| Tavassoli et al, 1989 | 52 | 21.2 | 11.6–33.0 | Southern | normal breast / TK, DHFR | 3 | – |
| Tsuda et al, 1989 | 176 | 4.0 | 1.8–7.6 | Slot Blots | normal breast / c-mos, PE4b-TGH2 | 3 | – |
| Zhou et al, 1989 | 157 | 6.4 | 3.0–10.1 | South/Slot | not specified / β-globin, INT-2 | 2 | + |
| Brouillet et al, 1990 | 140 | 22.9 | 16.4–30.1 | Southern | not specified / c-mos, β-globin | 2 | – |
| Meyers et al, 1990 | 99 | 1.0 | 0.1–4.6 | Southern | normal breast, placenta/thyroglobulin | 2 | – |
| Munzel et al, 1991 | 30 | 50.0 | 31.7–65.4 | Southern | not specified / not specified | – | + |
| Berns et al, 1992[a] | 282 | 20.0 | 15.6–24.9 | Southern | not specified / IGF-1-R | 3 | + |
| Berns et al, 1992[b] | 1052 | 17.2 | 15.0–19.5 | Southern | not specified / IGF-1-R | 2 | – |
| Borg et al, 1992 | 311 | 8.4 | 5.7–11.8 | Slot Blots | not specified / C-mos | 2 | + |
| Roux-Dosseto et al, 1992 | 65 | 24.6 | 15.1–35.5 | Southern | PBLs / β-globin | 2 | + |
| Kreipe et al, 1993 | 60 | 33.3 | 22.0–45.1 | Southern | normal breast / γ-interferon receptor | 1.8 | – |
| Nagayama et al, 1993 | 114 | 7.0 | 3.3–12.7 | Southern | normal breast / not specified | – | – |
| Ottestad et al, 1993 | 89 | 1.1 | 0.1–5.1 | Southern | not specified / COLIA2 | 3 | + |
| Scorilas et al, 1993 | 62 | 27.4 | 17.2–38.7 | Southern | normal breast / β-actin | – | + |
| Watson et al, 1993 | 154 | 7.0 | 3.3–11.1 | Southern/PCR | normal breast / c-mos | 2 | – |
| Yamashita et al, 1993 | 77 | 11.7 | 5.8–20.0 | Southern | PBLs / β-actin | – | – |
| Bieche et al, 1994 | 122 | 23.0 | 16.1–30.8 | Southern | PBLs / c-mos | 2 | – |
| Champeme et al, 1994 | 81 | 37.0 | 26.7–47.3 | Southern | PBLs / c-mos, β-globin | 2 | + |
| Harada et al, 1994 | 109 | 27.5 | 19.6–36.1 | Southern | normal breast / APO-B | – | – |
| Contegiacomo et al, 1995 | 54 | 5.7 | 1.5–14.0 | Southern | not specified / TGF-α | 2 | – |
| Lonn et al, 1995 | 162 | 16.1 | 11.0–22.2 | PCR | Tissue plasminogen activator gene | 4 | – |
| Scorilas et al, 1995[a] | 62 | 27.4 | 17.2–38.7 | Southern | not specified / β-actin | – | + |

amp = amplification; CI = confidence interval; + = present/adequate; –= not present/inadequate; PBLs = peripheral blood lymphocytes; COLIA2 = collagenI pro α-2 chain; [a]Not included in estimation of overall frequency of gene amplification because data for the same patients were given in an earlier report

*© 2000 Cancer Research Campaign*

or recurrence, as a function of *c-myc* amplification status, were reported, or could be derived, in six studies (Varley et al, 1987; Tsuda et al, 1989; Berns et al, 1992a; Borg et al, 1992; Yamashita et al, 1993; Lonn et al, 1995).

### Methodologic assessment

The studies were evaluated for the methods, analytic controls, and thresholds utilized to determine the frequency of *c-myc* amplification (Table 1). The primary sources of DNA controls were peripheral blood lymphocytes (PBLs) or normal breast tissue. Studies using PBLs exhibited a somewhat higher frequency of *c-myc* amplification (24.3%) and lower degree of variability (CV = 18.6%) than those using normal breast tissue (mean = 15.8%, CV = 25.1%), although the difference was not significant, $P = 0.180$. Most studies used *c-mos*, β-*globin*, or β-*actin* as normal gene controls for amplification. A two-fold increase was the most common amplification threshold, used in 65% of the 23 studies reporting a threshold. Most studies had adequate sample sizes, with 90% studying more than 50 patients, and 62% more than 100. A sample size of 50 gives reasonable precision for estimates of the proportion of *c-myc*-amplified tumours, with 95% confidence intervals in the range of ± 10–15 percentage points for most of the observed values of prevalence of amplification. The low percentage of small studies suggests that lack of statistical precision is unlikely to be a major source of heterogeneity in this meta-analysis. Only 41% of studies provided a description of the study population that is adequate for deriving clinically useful inferences; many did not indicate the prevalence of metastatic disease or whether treatment was given prior to surgery.

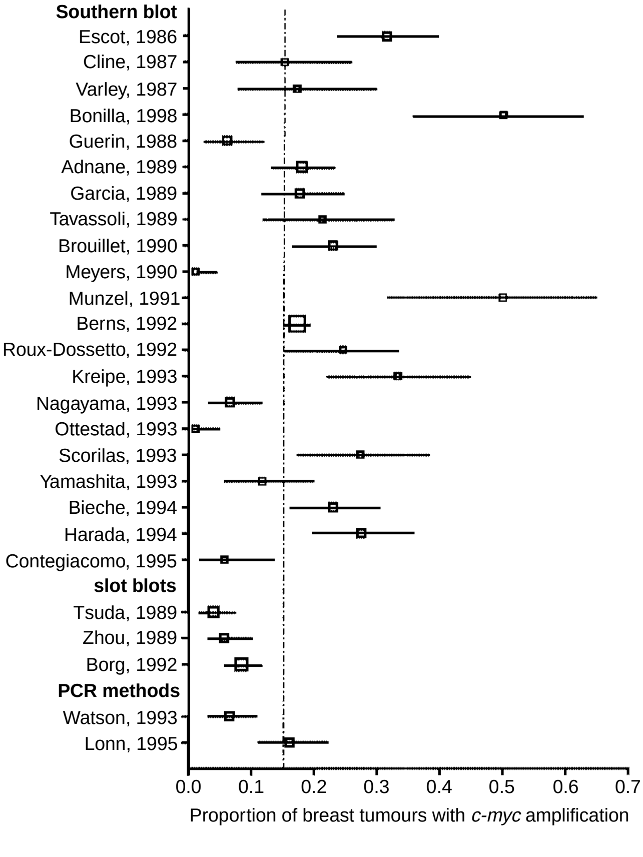

**Figure 1** Frequency of *c-myc* amplification in breast tumors by study and method. Data represent the proportion of tumours with *c-myc* amplification (boxes), and the 95% confidence interval around the proportion (bars). Box sizes represent the relative sample size for each study. The dotted line represents the weighted average over all studies for the proportion of *c-myc*-amplified tumours. Studies are grouped according to the method used to determine *c-myc* amplification. PCR = polymerase chain reaction

### Proportion of *c-myc*-amplified tumours

The proportion of *c-myc*-amplified breast tumours ranged from 1–50% (Figure 1). The estimate of the pooled average frequency of *c-myc* amplification was (15.7% (95% CI = 12.5–18.8%), based on the weighted average of 26 studies, comprising 3797 patients. However, there was considerable heterogeneity across the studies ($\chi^2 = 220.89$, df = 25, $P < 0.001$), indicating that the pooled average is an imprecise estimate of the amplification frequency to be expected in any study. Some of the heterogeneity could be due to differences in assay sensitivity and methods. Southern blotting was the only method used in more than three studies, with an overall frequency of 17.0% (95% CI = 13.5–20.5%). Slot blotting and PCR methods demonstrated a lower level of amplification with frequencies of 6.7% (95% CI = 0.1–14.3%) and (11.4% (95% CI = 0.4-22.4%), respectively. The two studies that employed two methods (Tsuda et al, 1989; Watson et al, 1993), Southern blotting and either slot blotting or PCR, for determining gene amplification indicated that both methods produced frequencies consistent with one another, although the individual values were not provided.

### Clinical associations of *c-myc* amplification

### *Association of* c-myc *amplification with known prognostic factors*

There was no significant heterogeneity across studies in the association between *c-myc* and any of the prognostic factors, except for age. Thus, it was valid to pool the estimates across studies for each of the remaining six prognostic factors. Poor histopathological grade, positive lymph node status, and negative progesterone receptor status were each associated with a significantly greater probability of *c-myc* amplification, while amplification was less likely in tumours from post-menopausal women (Table 2). Although we combined tumours with histopathological grades I and II in the above analysis, there was a trend toward increasing frequency of *c-myc* amplification with increasing grade, i.e. 12.5% for grade I, 20.3% for grade II, and 31.4% for grade III tumours. The association between lymph node status and *c-myc* amplification did not vary according to the threshold used to define amplification (data not shown). Because the association between *c-myc* and each of the other prognostic factors was evaluated in relatively few studies, the impact of the threshold for amplification could not be assessed.

### *Association with disease recurrence and overall survival*

The pooled estimates indicate that patients with *c-myc*-amplified tumours are approximately twice as likely as those with a normal level of *c-myc* to suffer disease recurrence or die (Tables 3A and 3B, respectively). For both disease-free (DFS) and overall survival (OS), there was no significant heterogeneity of HRs across the six studies. Exclusion of the two studies (Yamashita et al, 1993; Lonn et al, 1995), where HRs were estimated from the *P*-values, still resulted in approximately two-fold increases in risk. As with the individual prognostic factors, there was relatively little variation in

© 2000 Cancer Research Campaign

**Table 2** The association of c-myc amplification with breast cancer prognostic factors

| Prognostic factor | Studies (patients) | Rate ratio (95% CI) | RR $P$-value[a] | Test of homogeneity $\chi^2$ (df) | Test of homogeneity $P$-value[a] |
|---|---|---|---|---|---|
| Lymph node (+ vs –) | 13 (1551) | 1.24 (1.11–1.38) | 0.001 | 13.15 (12) | 0.379 |
| Oestrogen receptor (– vs +) | 8 (1712) | 1.13 (0.93–1.37) | 0.227 | 4.03 (7) | 0.683 |
| Progesterone receptor (– vs +) | 7 (1636) | 1.27 (1.08–1.49) | 0.004 | 7.64 (6) | 0.270 |
| Age (≥ 50 vs < 50 years) | 6 (1055) | [b] | [b] | 13.03 (5) | 0.023 |
| Tumor grade (III vs I/II) | 6 (551) | 1.61 (1.19–2.17) | 0.002 | 6.99 (5) | 0.222 |
| Menopausal status (post vs peri/pre) | 4 (697) | 0.82 (0.69–0.97) | 0.021 | 2.15 (3) | 0.547 |
| Tumour size (≥ 2 cm vs < 2 cm) | 4 (570) | 1.10 (0.95–1.27) | 0.219 | 2.10 (3) | 0.557 |

RR = rate ratio, i.e. the relative probability of amplified *c-myc* in tumours from patients in the first vs second category of the prognostic factor; CI = confidence interval; df = degrees of freedom for test of homogeneity; [a]Two-sided; [b]Estimates of the association between *c-myc* and age were not pooled across studies because there was significant heterogeneity among the estimates

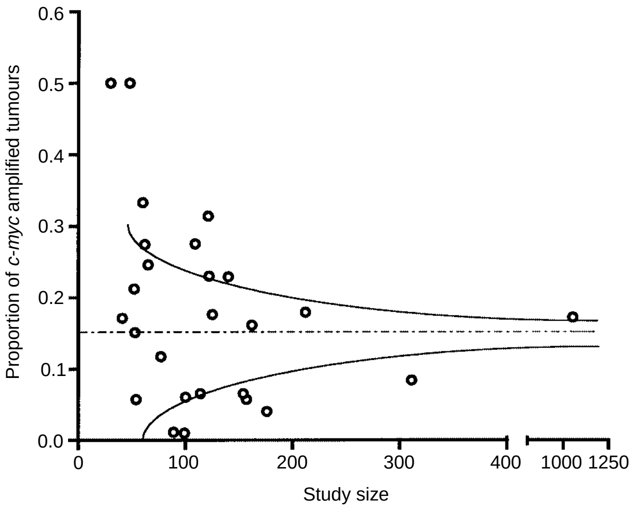

**Figure 2** Funnel plot of studies of *c-myc* amplification in breast tumours. Data represent the proportion of tumours exhibiting *c-myc* amplification in each study plotted against the sample size of the study. The dotted line represents the weighted average over all studies for the proportion of *c-myc*-amplified tumours. The solid curved lines are an approximation of an idealized 'funnel shape' expected in the absence of publication bias the threshold used to define *c-myc* amplification, so its influence on associations with survival could not be assessed.

### Publication bias

To evaluate publication bias (i.e. the phenomenon of studies with null results being less likely to be published) we generated a funnel plot of the reported frequency of *c-myc* amplification in each study plotted against the corresponding sample size (as a surrogate for the precision of the study). It is expected that the amount of scatter around the true mean frequency should decrease as the study sample sizes increase. The presence of publication bias would be indicated by a void in the lower left-hand corner of the funnel plot, suggesting a lack of small published studies presenting a low frequency of amplification (Dickersin et al, 1992). Although this method is subjective, Figure 2 suggests that a significant publication bias is unlikely in studies of *c-myc* amplification frequencies of less than 10%, with four of these studies having a sample size ≤ 100, one of which had a sample size of 54 (Figure 2).

### DISCUSSION

### Amplification of *c-myc*: prevalence in breast cancer

Our results suggest that approximately one in six breast cancers will display amplification of the *c-myc* gene. Because of the heterogeneity in individual study-specific estimates of *c-myc* amplification, this estimate may be less precise than suggested by the calculated 95% confidence interval of 12.5-18.8%. Due to the many potential technical and patient-related sources of variability that could not be controlled in our analysis, such heterogeneity is not surprising. Nevertheless, these data indicate that *c-myc* amplification is likely to occur only slightly less frequently than HER2/neu overexpression, a marker of apparent prognostic relevance in breast cancer.

A number of factors may contribute to heterogeneity of results, including tumour sampling variability, assay methodology, and patient populations. The amount and type of tumour material sampled may vary according to the surgical procedure and the size of the tumour. With current diagnostic trends (fortunately) shifting toward smaller tumours, research based on 'convenience' samples may be over-represented by tumours large enough to provide sufficient tissue for genetic analyses. In one study (Borg et al, 1992) the analysed samples were noted to be only 10–15% of all breast tumours diagnosed during that period. Sampling artifacts may also be related to the amount and location of normal control tissue taken at surgery, and perhaps, the area within the tumour that was sampled. Previous studies have demonstrated that expression of *c-myc* varies within the tumour, and that *c-myc* amplification can be seen in tissue at the leading edge of tumours (Watson et al, 1996).

Until recently, amplification of the *c-myc* oncogene has been evaluated by three principal methods: polymerase chain reaction, slot blotting, and Southern blotting, with the latter being the most widely utilized. These differing methodologies may have contributed to the inconsistency of the reported values of *c-myc* amplification, as they evaluate a mixed population of tumour and non-tumour cells. The future more widespread use of technologies that alleviate this problem, including fluorescence in situ hybridization (FISH), should allow for a more accurate assessment of gene amplification, deletions, and translocations by evaluating gene alteration at the single-cell level. FISH would also provide valuable information about the incidence of amplification in various cell types, particularly in tumour samples that may exhibit genetic heterogeneity. The usefulness of this technique in assessing *c-myc* genetic alterations has been demonstrated in other

 © 2000 Cancer Research Campaign

**Table 3** Study-specific and pooled hazard ratio (HR) estimates of (**A**) disease-free survival and (**B**) overall survival in breast cancer patients with *c-myc*-amplified tumours vs non-amplified tumours.

| Reference (*n*) | Hazard ratio | 95% CI | Weight[a] | Adjustment variables |
|---|---|---|---|---|
| **A** | | | | |
| Yamashita et al, 1993 (77)[b] | 3.02 | 0.72–12.59 | 1.88 | LN, ER, size, menopausal status, erbB2, Rb, p53, int-2, NDP Kinase |
| Varley et al, 1987 (35)[b] | 4.11 | 0.48–35.43 | 0.83 | Grade |
| Tsuda et al, 1989 (176) | 4.42 | 1.35–14.48 | 2.72 | LN, size, erbB2, Tx |
| Berns et al, 1992 (282) | 1.80 | 1.30–2.60 | 28.41 | LN, ER, size |
| Borg et al, 1992 (311) | 2.09 | 0.83–5.26 | 4.51 | LN, ER, PR, size |
| Lonn et al, 1995 (122)[b] | 2.15 | 0.66–7.01 | 2.76 | None |
| Pooled estimate | 2.05 | 1.51–2.78 | Test of homogeneity, $\chi^2$ = 3.47 (5 df), $P$ = 0.63 | |
| **B** | | | | |
| Yamashita et al, 1993 (77)[b] | 4.79 | 1.45–15.79 | 2.70 | LN, ER, size, menopausal status, erbB2, Rb, p53, int-2, NDP Kinase |
| Varley et al, 1987 (35)[b] | 1.99 | 0.36–10.90 | 1.32 | None |
| Tsuda et al, 1989 (176) | 1.50 | 0.53–4.19 | 3.64 | LN, size, erbB2, Tx |
| Berns et al, 1992*a* (282) | 1.40 | 1.00–2.20 | 18.81 | LN, ER, size |
| Borg et al, 1992 (311) | 2.00 | 1.00–3.90 | 8.61 | LN, ER, PR, size |
| Lonn et al, 1995 (122)[b] | 2.07 | 0.68–6.34 | 3.08 | None |
| Pooled estimate | 1.74 | 1.27–2.39 | Test of homogeneity, $\chi^2$ = 4.0 (5 df), $P$ = 0.55 | |

CI = confidence interval; LN = lymph node metastases; ER = oestrogen receptor status; PR = progesterone receptor status; Tx = treatment; [a]weight = 1/variance [ln (HR)]; [b]HR (hazard ratio) derived by authors of this meta-analysis as described in Methods cancers, including prostatic and haematopoeitic malignancies (Jenkins et al, 1997). It will also be of use for future studies to utilize in situ hybridization and immunohistochemistry (IHC), together with FISH, to provide a more comprehensive view of amplification relative to overexpression, particularly since antibodies suitable for IHC are now commercially available.

Finally, the patient populations within each study may bias the sample toward higher or lower prevalence of gene amplification, or toward better or worse prognoses. Translating basic research on the biology and function of tumour-associated genes to their evaluation in clinical populations requires attention to aspects of population-based studies that can complicate interpretation of clinical relevance. Details of tumour stage, metastatic prevalence, adjuvant therapy, and average length of patient follow-up were frequently omitted. The time period over which patients were accrued may also be relevant, as it may identify differences in diagnostic or treatment practices.

### Amplification of *c-myc*: role as an independent prognostic factor

Patients with *c-myc* amplified tumours had an approximate two-fold increase in risk of relapse or death. For *c-myc* to be useful as an independent prognostic factor, it should not be merely a surrogate for one or more established prognostic factors. Although *c-myc* amplification appears to be somewhat more common in tumours with more aggressive phenotype (Table 2), it retained its independent prognostic value in the four studies where survival analyses were adjusted for lymph node metastasis, tumour size and/or ER status. Thus, it is likely that *c-myc* has prognostic value independent of these factors. Adjustment for tumour grade was included in only one of the six survival studies, and this meta-analysis has shown a significant correlation between high tumour grade and *c-myc* amplification (RR = 1.61, Table 2). Thus, we cannot rule out the possibility that the association between *c-myc* and survival reflects, to some degree, a confounding influence arising from the association between *c-myc* and grade. However, for this association to be due entirely to confounding by grade would require that the association between amplification and grade be stronger (higher HR) than that observed between amplification and survival. Thus, it is unlikely that grade explains all or most of the association between *c-myc* and relapse or survival.

Although survival was evaluated in only six studies, the above data suggest that amplified *c-myc* may have significant value as an independent prognostic factor. Table 3 shows that the magnitude of the pooled HR estimates for DFS and OS are heavily influenced by the large weight (small variance) of Berns et al (1992*a)*. However, the other five studies have individual HRs that are similar to or larger than this, suggesting that the large weight of the latter contributes toward a more conservative estimate. There is no obvious source of bias that would produce this association with survival as an artifact. Nevertheless, the retrospective nature of the studies requires that this association be viewed as the starting point for more rigorous assessment rather than an established prognostic factor. Additional studies should optimally be embedded in randomized clinical trials to ensure uniform ascertainment and control of confounding factors. Such studies could also allow distinction between prognostic and predictive factors (Hayes et al, 1998).

Little has been published about the effect of *c-myc* on treatment response, particularly in breast cancer. New predictive factors are needed, because conventional histopathologic factors do not accurately predict the likelihood that an individual patient will respond to cytotoxic chemotherapy. This is a possible area for future investigation, as some studies have implicated expression of *c-myc* in resistance to cisplatin-based chemotherapy in multiple tumour types (Walker et al, 1996), in MDR1 expression in rhabdomyosarcoma cell lines (Prados et al, 1996), and in resistance to 5-fluorouracil and cisplatin in squamous cell carcinomas of the head and neck (Riva-Lavielle, 1994). A rigorous study, designed specifically to address the relationship between *c-myc* amplification and drug and hormone resistance in breast cancer, may provide valuable insight into more effective treatment strategies.

*© 2000 Cancer Research Campaign*

### Amplification of *c-myc* in relation to other oncogenes

It has been hypothesized that *c-myc* amplification is a marker for genetic instability, and its permissive effect on downstream gene amplification, or its interaction with co-factor mutations may enhance the likelihood of disease progression to a more aggressive phenotype. A study by Janocko et al (1995) found a preferential sequence for gene amplification in breast cancer, with *c-myc* most often occurring first, followed by *c-erbB-2*. Sierra et al (1999) demonstrated in breast cancer patients that Bcl-2 overexpression was strongly associated with lymph node metastasis, but only when *c-myc* was co-expressed. Caspase 9 is a downstream co-factor in *myc*-dependent tumourigenesis and progression. Deletion of the gene encoding caspase 9 has been shown to block *myc*-induced apoptosis and to further promote its oncogenic transformation (Soengas et al, 1999). These data are also consistent with earlier reports implicating the 1p34–36 chromosomal region, where the caspase 9 gene maps, as a locus of frequent deletion in human tumours (Bieche et al, 1994; Mertens et al, 1997). These studies provide further support for the examination of co-factor mutations in the context of *c-myc* amplification as a means of defining the prognostic value of this oncogene.

### ACKNOWLEDGEMENTS

This work was supported by a grant from the National Cancer Institute (NCI R01 CA 72460: Dr RB Dickson), and a grant from the National Institute of Aging (NIA R01 AG 1496: Dr RB Dickson). The authors of this manuscript would like to thank Drs Edward Rosfjord and Michael Johnson for their valuable discussion and critical review. We would like to thank Dr Fabio Leonessa for his helpful input.

### REFERENCES

Adnane J, Gaudray P, Simon MP, Simony-Lafontaine J, Jeanteur P and Theillet C (1989) Proto-oncogene amplification and human breast tumor phenotype. *Oncogene* **4**: 1389–1395

Alitalo K, Schwab M, Lin CC, et al (1983) Homogeneously staining chromosomal regions contain amplified copies of an abundantly expressed cellular oncogene (c-myc) in colon carcinoma. PNAS **80**: 1707–1711

Berns EMJJ, Klijn JGM, van Puten WLJ, van Staveren IL, Portengen H and Foekens JA (1992*a*) C-myc amplification is a better prognostic factor than HER2/neu amplification in primary breast cancer. *Cancer Res* **52**: 1101–1113

Berns EMJJ, Klijn JGM, van Staveren IL, Portengen H, Noordegraaf E and Foekens JA (1992*b*) Prevalence of amplification of the oncogenes c-myc, HER2/neu, and int-2 in one thousand human breast tumors: Correlation with steroid receptors. *Eur J Cancer* **28**: 697–700

Bieche I, Champeme MH and Liderau R (1994) A tumor suppressor gene on chromosome 1p32-pter controls the amplification of MYC family genes in breast cancer. *Cancer Res* **54**: 4274–4276

Bonilla M, Ramirez M, Lopez-Cueto J and Gariglio P (1988) In vivo amplification and rearrangement of c-myc oncogene in human breast tumors. *J Natl Cancer Inst* **80**: 665–671

Borg A, Baldetorp B, Ferno M, Olsson H and Sigurdsson H (1992) c-myc is an independent prognostic factor in postmenopausal breast cancer. *Int J Cancer* **51**: 687–691

Breslow NE and Day NE (1987) *Statistical methods in cancer research, Vol II*. IARC Scientific Publication 94–2789, pp 110–112. IARC: Lyon, France

Brouillet JP, Theillet C, Maudelonde T, Defrenne A, Simony-Lafontaine J, Sertour J, Pujol H, Jeanteur P and Rochefort H (1990) Cathepsin D assay in primary breast cancer and lymph nodes: Relationship with c-myc, c-erbB-2, and int-2 oncogene amplification and node invasiveness. *Eur J Cancer* **26**: 437–441

Champeme MH, Bieche I, Hacen K and Lidereau R (1994) Oncogene amplification per se: an independent prognostic factor in human breast cancer. *Mol Carcinog* **11**: 189–191

Cline M, Battifora H and Yokota J (1987) Proto-oncogene abnormalities in human breast cancer: Correlations with anatomic features and clinical course of disease. *J Clin Oncol* **5**: 999–1006

Contegiacomo A, Pizzi C, Demarchis L, Alimandi M, Delrio P, Dipalma E, Petrella G, Ottini L, French D, Frati L and Bianco AR (1995) High cell kinetics is associated with amplification of the Int-2, Bcl-1, Myc and ErbB-2 protooncogenes and loss of heterozygosity at the DF3 locus in primary breast cancers. *Int J Cancer* **61**: 1–6

Courjal F, Cuny M and Simony-Lafontaine J (1997) Mapping of DNA amplification at 15 chromosomal localizations in 1875 breast tumors: Definition of phenotypic groups. *Cancer Res* **57**: 4360–4367

Dalla-Farera R, Wong-Staal F and Gallo RC (1985) One gene amplification in promyelocytic leukaemaia cell line HL-60 and primary leukaemic cells of the same patient. *Nature* **299**: 61–63

Dang CV (1999) *C-myc* target genes involved in cell growth, apoptosis, and metabolism. *Mol Cell Biol* **19**: 1–14

Dickersin K and Berlin JA (1992) Meta-analysis: state-of-the-science. *Epidemiologic Rev* **14**: 154–176

Edwards PA, Ward JL and Bradbury JM (1988) Alteration of morphogenesis by the c-myc oncogene in transplants of mammary gland. *Oncogene* **2**: 407–412

Escot C, Theillet C, Linderiau R, Spyratos F, Champeme MH, Gest J and Callahan R (1986) Genetic alteration of the c-myc protooncogene (MYC) in human primary breast carcinomas. *Proc Natl Acad Sci USA* **83**: 4834–4839

Fleiss JL (1981) *Statistical methods for rates and proportions*. John Wiley and Sons: New York

Garcia I, Dietrich PY, Aapro M, Vauthier G, Vadas L and Engel F (1989) Genetic alteration of c-myc, c-erbB-2, and c-Ha-ras protooncogenes and clinical associations in human breast carcinomas. *Cancer Res* **49**: 6675–6679

George SL and Desu MM (1974) Planning the size and duration of a clinical trial studying the time to some critical event. *J Chronic Dis* **27**: 15–24

Guerin M, Barrois M, Terrier MJ, Spielmann M and Riou G (1988) Overexpression of either c-myc or c-erbB-2/neu proto-oncogenes in human breast carcinomas: correlation with poor prognosis. *Oncogene Res* **3**: 21–31

Harada Y, Katagiri T, Ito I, Akiyama F, Sakamoto G, Kasumi F, Nakamura Y and Emi M (1994) Genetic studies of 457 breast cancers, clinicopathologic parameters compared with genetic alterations. *Cancer* **74**: 2281–2286

Hayes DF, Trock B and Harris AL (1998) Assessing the clinical impact of prognostic factors: when is ‘statistically significant’ clinically useful? *Breast Cancer Res Treat* **52**: 305–319

Janocko LE, Lucke JF and Groft DW, et al (1995) Assessing sequential oncogene amplification in human breast cancer. *Cytometry* **21**: 18–22

Jenkins RB, Qian J, Lieger MM and Bostwick DG (1997) Detection of c-myc oncogene amplification and chromosomal anomalies in metastatic prostatic carcinoma by fluorescence in situ hybridization. *Cancer Res* **57**: 524–31

Kleinbaum DG, Kupper LL and Morgenstern H (1982) *Epidemiologic Research*, p 359. Van Nostrand Reinhold Co: New York

Kreipe H, Fischer L, Felgner J, Heidorn K, Mettler L and Parwaresch R (1993) Amplification of c-myc, but not c-erbB2 is associated with high proliferative capacity in breast cancer. *Cancer Res* **53**: 1956–1961

Leder A, Pattengale PK, Kuo A, Stewart T and Leder P (1986) Consequences of widespread deregulation of the c-myc gene in transgenic mice: multiple noeoplasms and normal development. *Cell* **45**: 485–495

Little CD, Nau MM and Carney DN (1985) Amplification and expression of the *c-myc* oncogene in human lung cancer cell lines. *Nature* **306**: 194–196

Lonn U, Lonn S, Nilsson B and Stenkvist B (1995) Prognositic value of the erbB-2 and myc amplification in breast cancer imprints. *Cancer* **75**: 2681–2687

Mertens F, Johansson B, Hoglund M and Mitelman F (1997) Chromosomal imbalance maps of malignant solid tumors: a cytogenetic survey of 3185 neoplasms. *Cancer Res*. **57**: 2765–2780

Meyers S, O’Brien M, Smith T and Dudley J (1990) Analysis of the int-1, int-2, c-myc, and neu oncogenes in human breast carcinomas. *Cancer Res* **50**: 5911–5918

Munzel P, Marx D, Kochei H, Schauer A and Bock KW (1991) Genomic alterations of the c-myc proto-oncogene in relation to the overexpression of c-erbB-2 and KI-67 in human breast and cervix carcinomas. *J Cancer Res Clin Oncol* **117**: 603–607

Nagayama K and Watatani M (1993) Analysis of genetic alterations related to the development and progression of breast carcinoma. *Jpn J Cancer Res* **84**: 1159–1164

Nass SJ and Dickson RB (1997) Defining a role for *c-myc* in breast tumorigenesis. *Breast Cancer Res Treat* **44**: 1–22

© 2000 Cancer Research Campaign

Ottestad L, Andersen T, Hesland J, Skrede M, Tveit K, Nustad K and Borresen AL (1993) Amplification of c-erbB-2, int-2 and c-myc genes in node-negative breast carcinomas. *Acta Oncologica* **3**: 289–294

Prados J, Melguizo C, Fernandez A, Aranega AE, Alvarez L and Aranega A (1996) Inverse expression of mdr 1 and c-myc genes in a rhabdomyosarcoma cell line resistant to actinomycin D. *J Pathol* **180**: 86–89

Riva-Lavielle C (1994) Drug resistance, oncogenes, and anti-oncogenes in epithelial tumours. *Bull Cancer* **81**: 105s–111s

Roux-Dosseto M, Romain S, Dussault N, Desideri C, Piana L, Bonnier P, Tubiana N and Martin PM (1992) C-myc gene amplification in selected node-negative breast cancer patients correlates with high rate of early relapse. *Eur J Cancer* **28A**: 1600–1604

Scorilas A, Yotis J, Gouriotis D, Keramopoulos A, Ampela K, Trangas T and Talieri M (1993) Cathepsin-D and c-erb-B2 have an additive prognostic value for breast cancer patients. *Anticancer Res* **13**: 1895–1900

Scorilas A, Yotis J, Stravolemos K, Gouriotis D, Keramopolous A, Ampela K, Talieri M and Trangas T (1995) c-erbB-2 overexpression may be used as an independent prognostic factor for breast cancer patients. *Anticancer Res* **15**: 1543–1548

Sierra A, Castellsague X, Escobedo A, Moreno A, Drudis T and Fabra A (1999) Synergistic cooperation between c-Myc and Bcl-2 in lymph node progression of T1 human breast carcinomas. *Breast Cancer Res Treat* **54**: 39–45

Soengas MS, Alarcon RM, Yoshida H, Giaccia AJ, Hakem R, Mak TW and Lowe SW (1999) Apaf-1 and Caspase-9 in p53 dependent apoptosis and tumor inhibition. *Science* **284**: 156–159

Tavassoli M, Quirke P, Farzaneh F, Lock NJ, Mayne LV and Kirkham N (1989) C-erbB-2/cerbA coamplification indicative of lymph node metastais, and c-myc amplification of high tumor grade, in human breast carcinoma. *Br J Cancer* **60**: 505–510

Trock BJ, Leonessa F and Clarke R (1997) Multidrug resistance in breast cancer: a meta-analysis of MDR1/gp 170 expression and its possible functional significance. *J Natl Cancer Inst* **13**: 917–931

Tsuda H, Hirahashi S, Shiomosata Y, Hirota T, Tsugane S, Yamamoto H, Miyajima N, Toyoshima K, Yamamoto T, Yokota J, Yoshida T, Sakamura H, Terada M and Sugimura T (1989) Correlation between long-term survival in breast cancer patients and amplification of two putative oncogene-coamplification units hst-1/int-2 c-erbB-2/ear-1. *Cancer Res* **49**: 3104–3108

Varley JM, Swallow JE, Brammar WJ, Whittaker, JL and Walker RA (1987) Alteration to either c-erbB-2 (neu) or c-myc proto-oncogenes in breast carcinomas correlate with poor short-term prognosis. *Oncogene* **1**: 423–430

Varmus HE (1984) The molecular genetics of cellular oncogenes. *Annu Rev Genet* **15**: 553–612

Walker TL, White JD, Esdale WJ, Burton MA and De Cruz EE (1996) Tumour cells surviving in vivo cisplatin chemotherapy display elevated c-myc expression. *Br J Cancer* **73**: 610–614

Watson PH, Safneck JR, Lee K, Dubik D and Shiu RPC (1993) Relationship of c-myc amplification to progression of breast cancer from in situ to invasive tumor and lymph node metastasis. *J Natl Cancer Inst* 902–907

Watson PH, Singh R and Hole A (1996) Influence of c-myc on the preogression of human breast cancer. *Curr Topic Microbiol Immunol* **213**: 267–283

Yamashita H, Kobayashi S, Iwase H, Itoh Y, Kuzushima T, Iwata H, Itoh K, Naito A, Yamashita T, Masaoka A and Kimura N (1993) Analysis of oncogenes and tumor suppressor genes in human breast cancer. *Jpn J Cancer Res* **84**: 871–878

Zhou DJ, Ahuja H and Cline MJ (1989) Proto-oncogene abnormalities in human breast cancer: c-erbB-2 amplification does not correlate with recurrence of disease. *Oncogene* **4**: 105–108

*© 2000 Cancer Research Campaign*
